# Abdominal Surgery

**Published:** 1886-12-20

**Authors:** Arch Dixon


### ABDOMINAL SURGERY.

BY ARCH DIXON.

Read before the McDowell Medical Society at Hopkinsville, Ky., May 15, 1886.

Until a comparatively recent date, the abdominal cavity was the "terra incognita" of the surgeon, and intra peritoneal surgery was unrecognized except under the direct necessity, and no surgeon was brave enough to encroach upon its domain, save perhaps for the relief of a strangulated hernia. Indeed, had things which are now done every day been so much as even hinted at then, the hints would have been considered to be simply the thoughts of an enthusiast, and any attempt to carry them into effect would have been regarded as insane. Surgeons are now beginning to understand that laparotomy is not such a dreadful thing after all. That it is a capital operation all must admit, but so are many others that no surgeon hesitates to perform, and the idea is rapidly becoming a thing of the past that the peritoneum is a structure which must not be touched. In those cases arising from strictures of the pharynx, æsophagus and cardiac orifices of the stomach, gastrotomy, combined with dilation of the orifices of the stomach, as advocated by Prof. Toretta, of Bologna, has developed a means of affording relief, though not of cure, for a class of diseases, the agony of which, ere their course was run to its unaided bitter end, was beyond description, and oftentimes beyond all the strength of human endurance, and has found for the sufferers a form of euthanasia, at any rate. which enables them to die in peace.

I have recently had a case in which my patient suffered untold agony and died a most horrible death simply because he could not be induced to submit to an operation. There is no longer any hesitancy in regard to the surgical treatment of obstruction as occurring in the intestinal canal. Some of which, in the acute form, give rise to symptoms so identical with those of strangulated hernia that if any external manifestations were present a surgeon would feel bound to explore it, open the body, search for the obstruction and if possible remove it ; the same may be said of cases more chronic, with acute symptoms suddenly added, though the prospect of successful operative interference is considerably diminished in these cases, yet it is nevertheless the duty of the surgeon to make it. In cases of wounds of the abdomen, either gun-shot or stab, complicated by a wound of the intestines, the indications are equally as plain and as positive.

If through a wound in the abdominal wall, unwounded intestines be found to protrude, the line of treatment is plain enough. Carefully and thoroughly cleanse the intestine and return it well into the abdominal cavity, and so stitch up the wound in the latter, under antiseptic precautions and dressings, as to place it in the most favorable conditions to heal, and you may reasonably hope to be successful. Should the intestine be wounded, remedy the wound before replacing the bowel, if possible making it leak-tight; this can only be done by turning the peritoneal coat well in, so as to bring together a sufficient amount of peritoneal surface, sewing the rounded edges so closely and so firmly together as to insure their withstanding the pressure of even liquid contents in their passage through the canal, and for this I would recommend Czerney's Modification of Dr. Lembert's Stitch. A case so treated will also generally make a favorable recovery. Without this operative interference your patient will surely die; with it there may happen that recovery uninterrupted takes place, or that the contents of the intestine escapes into the peritoneal cavity producing a speedy and fatal issue, unless the cavity is at once opened up and washed out; or perchance the edges of the intestinal wound may become adherent to those of the wound in the abdomen, thus forming a fecal fistula, which, however unpleasant this may be, is still preferable to death, and moreover by a suitable operation this fecal fistula may be closed. In occlusion of the duct of the gall bladder, either by inflammation or by billiary calculi, the operation of cholecystotomy has been performed so often and with so much success that it may now be classed among the recognized surgical procedures. In no portion of the domain of abdominal surgery has greater progress been effected than in that of renal surgery. For "floating kidney," for "circum renal cysts," for "abscess of the kidney," for "calculus," impacted in the kidney, for carcinama and sarcoma of the kidney, surgical aid has been successfully invoked, until now the kidney has come to be considered as fully subject to the surgeon, the operations of nephrectomy and nephrotomy being of almost daily occurrence.—(Wheelhouse in *British Med. Journal.*)

It may be asked whether commencing or existing peritonitis contra-indicates laparotomy. Fortunately the successes and experiences of Schramm, Boully, Isreal, Schrœder, Kieth, Wells and Dennis, of New York, and others in this country, have already given a negative answer to this question. Peritonitis occurs but rarely after operations compared with its frequency after accidents, and it has in a large number of cases been shown that it rapidly subsides when due to traumatism, after an operation has been performed the abdomen washed and sponged out and drainage established. As regards the operation itself, Bergman says that it is a secondary question to that of diagnosis ; that is to say, he does not now hesitate to perform laparotomy in cases in which the symptoms point to an early death, as he did a few years ago. Consequently, as these cases may imperatively demand an operation, the easiest and surest way to get out of the difficulty is to perform laparotomy. This establishes the diagnosis, and if an operation be demanded the first steps have already been taken. The risk to the patients is very much less when the operation is performed before a local peritonitis has become general.—(*Journal Am. Med. Ass'n.*)

Hueter, in his surgery, says that laparotomy, made in cases of traumatic injury to the contents of the abdominal cavity, under antiseptic precautions will absolutely prevent all symptoms of septic peritonitis and the greatest protection which can be afforded in traumatic injuries of the peritoneum is by prophylactic antiseptic laparotomy. The subject of laparotomy for diagnostic purposes was very tersely put by Mr. Lawson Tait, during his recent visit to this country, when on being asked for an opinion regarding the nature of an obscure abdominal tumor he said, "cut the patient open and find out." And it is interesting to know that the patient was cut open and saved by hysterectomy from almost certain death. In his address, as chairman of the surgical committee of the Medical Society of the County of Kings, delivered in October, 1885, Dr. Geo. R. Fowler says: But there is yet room for missionary work before men, and good men, too, can be induced to come out of their shell of conservatism, so called, and with a bold front help to break down the prejudices and misgivings based upon an ill-founded fear of the peritoneum and its behavior under the knife. Dr. Fowler divides the cases calling for explorative laparotomy into four classes. (1) Cases in which a diagnosis cannot be made without opening the abdomen and exposing the parts to the direct touch, or even the sight, of the surgeon, but in which further interference is thereby shown to be impracticable or uncalled for. (2) Cases in which a provisional diagnosis only can be made, unaided by abdominal incision, and in which but slight additional risk is incurred by an immediate and radically curative procedure, based upon the knowledge thus gained. (3) Cases in which a diagnosis has been made, but in which doubt exists as to the practicability of performing a radical operation ; and cases iu which the choice of the particular operation best adapted to the individual case must be decided upon after incision and exploration. (4) Cases in which the patient's life is in imminent peril, and in which it becoms imperatively necessary to at once locate the lesion threatening life, and to be prepared to act promptly upon the knowledge gained by opening the abdominal cavity. The cases included under the first class are those in which an operation would be impracticable or is uncalled for. It is well known that there are many cases of severe pelvic, or abdominal pain, in which a diagnosis is impossible without an operation, though the symptoms give a strong suspicion of chronic ovaritis or salpingitis. In some cases nothing can be found by an operation to account for the pain and other symptoms, and yet they disappear promptly after the operation. In cases under the second class a partial diagnosis has been made, sufficient with the symptoms to show that the patient's life is in danger and that something must be done. As illustrative of this class of cases, chronic ovaritis or salpingitis, with or without hydro or pyosalpinx, may be cited. In some of these cases, although attended with considerable difficulty in making a positive diagnosis, yet the suspicion amounts almost to a certainty. Here an opening sufficiently large to admit the index finger will clear up the doubt. The third division applies to cases in which a diagnosis has been made, but some doubt still exists as to the practicability of performing a radical operation. In this sense most cases of ovariotomy and hysterectomy may perhaps be looked upon as being, in some degree, exploratory operations, as easily seen by the number of cases in which an operation has been commenced, but abandoned on account of the high improbability of success. Hence the wisdom of always making the incision as if for simple explorative purposes; when, if the operation be found impracticable, the wound to be healed is not so large. Cases of uterine fibroma of small or medium size which, because of exhausting hemorrhage, demand interference, will require a preliminary explorative operation before a choice can be made between simple removal of the appendages or a hysterectomy. Of course, in the greater number of cases removal of the appendages is sufficient and perfectly practicable, but now and then a case is met with in which the only choice is between the more formidable operation and closure of the incision without completing the operation. The fourth and last class includes those cases in which the patient's life is in imminent danger, and something must be done at once both to locate the lesion and to apply means for its relief. And, strange to say, this is the class in which the so-called conservatism is most frequently hown. Illustrative cases of this class are those of rupture of an extra-uterine pregnancy, of rupture of the pregnant uterus with escape of the fœtus into the abdominal cavity, as reported by Plenio in a recent number of the *Centralblatt fur Gymnækologie*, in which the patient's life was saved. The successes of Bull, Hamilton and others with gun-shot wounds of the intestines are alone sufficient to warrant an explorative laparotomy when such cases occur, and in regard to operations for ruptured extra-uterine pregnancy we need only refer to the successes of Mr. Lawson Tait. The readiness with which the surgeon will have recourse to laparotomy will depend upon the confidence he may have in the relative innocuousness of the operation when properly performed, upon his own experience in abdominal surgery, and in his reliance upon his ability to control the conditions which the abdominal section may develop. I would by no means encourage an indiscriminate and hasty resort to laparotomy, but I can hardly conceive that an intelligent practitioner would come to the conclusion that a disturbance had taken place in the abdominal cavity of so profound a character as to induce him to incise its walls for its relief, without the result amply justifying the procedure. The condition developed may not be found as supposed, or it may prove to be of a character not susceptible of relief, but the operation will have had the result of clearing up a hitherto obscure condition, or of satisfying an operator that he has not denied to his patient any chance of relief; but if it is found to be of a nature susceptible of relief by performing the laparotomy, as I have said before, he has already taken the first step in the direction of securing that relief. From my point of view I am led to think that there is far more danger of operative interference being delayed until the period when it may be of avail has past, than that an unnecessary and hasty operation would be done.

